# The Fruits of the Harvest

**Published:** 1920-10-16

**Authors:** 


### The Fruits of the Harvest.

At this time of the year, hospitals are the glad recipients of quantities of fruit and vegetables given by church congregations and allotment associations. Even in London and other urban areas these gifts are numerous and valuable, while hospitals situated in the midst of agricultural districts are especially fortunate. The quantity of produce presented to such institutions in the week following the Harvest Thanksgiving Service is in some cases apt to be embarrassing, particularly if the articles are of a perishable nature. In such cases all available hands are at once employed in making jam, chutney, and pickles; whilst the hard fruit and vegetables are carefully stored away for winter use. As the hospitals generally receive the produce that has been exhibited at the Harvest Thanksgiving Services, and as such produce naturally includes the finest growths in each variety, the beneficiaries are favoured as much in quality as in quantity. One provincial hospital having been presented with a monster pumpkin, whose huge dimensions were of much interest but of some practical inconvenience, the opportunity was taken to exhibit the vegetable in the window of a friendly trader. Passers-by were then invited to guess the weight of the pumpkin at a penny a time, a prize being given for a correct guess; and in this way several pounds were raised for the hospital, in whose interest the vegetable was ultimately sold by auction.

